# Identification of C1QA as a prognostic marker and regulator of immunosuppressive neutrophils in early-stage lung adenocarcinoma through integrated bioinformatics analyses

**DOI:** 10.1007/s10238-025-01881-y

**Published:** 2025-10-31

**Authors:** Quynh Anh Nguyen, Hao Wang, Elizabeth Verghese, Joshua Iscaro, Daniel Steinfort, Jonathan McQualter, Steven Bozinovski

**Affiliations:** 1 Centre for Respiratory Science & Health, School of Health & Biomedical Sciences, RMIT University, Bundoora VIC, PO Box 71, Melbourne, 3083 Australia; 2 Department of Respiratory Medicine & Sleep Medicine, Royal Melbourne Hospital, Melbourne, Australia; 3 Faculty of Medicine, University of Melbourne, Parkville, Australia

**Keywords:** C1QA, C1q, Complement system, Neutrophils, LUAD, Lung adenocarcinoma

## Abstract

Neutrophils infiltrate lung tumours and can exhibit an immunosuppressive phenotype that promotes tumour growth, yet their regulation in early-stage lung cancer remains unclear. This study investigates molecular regulators of neutrophils in early-stage lung adenocarcinoma (LUAD) using publicly available gene expression datasets. An early-stage LUAD dataset (GSE31210) was analysed using CIBERSORTx to estimate neutrophil abundance. Weighted gene co-expression network analysis (WGCNA) identified the hub gene most correlated with neutrophil scores. Single-cell RNA sequencing (scRNA-seq) was used to determine the cellular source and regulatory mechanisms of this gene. A high neutrophil score was a negative prognostic factor, validated in independent datasets. WGCNA identified C1QA as the key gene linked to neutrophil abundance. scRNA-seq and immunofluorescence staining confirmed macrophages as the primary source of C1QA. Cell–cell communication analysis suggested C1QA interacts with neutrophils via complement receptors, contributing to an immunosuppressive tumour microenvironment. RT-qPCR showed C1QA expression correlated with IL-10 and TGFB1, markers of immunosuppression. C1QA is a poor prognostic marker in LUAD, potentially driving immunosuppressive tumour-associated neutrophils. Targeting C1QA and its pathway may offer a novel therapeutic strategy in early-stage LUAD.

**Supplementary Information:**

The online version contains supplementary material available at 10.1007/s10238-025-01881-y.

## Background

Lung cancer is the term referring to malignancies that originate in the lung parenchyma or within the bronchi [1]. It is the leading cause of cancer-related deaths worldwide [1]. About 52% patients with localised disease can survive up to 5 years. However, the survival rate drops significantly to below 5% if distant metastasis is diagnosed [2]. Non-small cell lung cancer (NSCLC) is the most common type of lung cancer. It accounts for 85% of all lung cancer diagnoses. Under the NSCLC category, lung adenocarcinoma (LUAD) is the dominant histological subtype, making up approximately 40% of all lung cancer cases [2, 3].

Several studies have highlighted the prognostic value of infiltrating immune cells in cancer, underscoring their active involvement in disease progression [4, 5]. Tumour-infiltrating immune cells are generally classified as facilitating either tumour suppression or tumour aggressiveness. A pro-inflammatory tumour microenvironment (TME) characterised with the abundance of CD8 T cells, is linked to improved clinical outcomes and better response to immunotherapy across several malignancies [6]. Conversely, the presence of myeloid-derived suppressor cells (e.g. neutrophils and macrophages) and regulatory T cells are reported to be immune suppressive and hinder tumour killing capability, leading to impaired immunotherapy efficacy and worse patient outcome [7, 8].

There is emerging evidence that neutrophils, when trafficking into tumours can acquire either a pro- or antitumour phenotype in various cancer types. The phenotype of these tumour-associated neutrophils (TANs) is dictated by distinct cytokines under the influence of the TME. Neutrophils are short-lived immune cells that readily undergo apoptosis. Therefore, identifying neutrophil subpopulations with distinct phenotypes can pose a challenge. Nonetheless, the evaluation of the intratumoural Neutrophil-to-Lymphocyte Ratio (iNLR), determined by quantifying the intratumoural CD66b + neutrophil to CD8 + T-cell ratio, has identified this cellular marker as an independent predictor of poor outcome regardless of tumour stage in patients with resectable NSCLC [9]. These findings are consistent with the presence of immunosuppressive neutrophil phenotype in the TME that directly blocks the actions of cancer killing cytotoxic T cells. In contrast with such studies, a number of studies have reported that the presence of neutrophils during the early stage of lung tumour development can contribute to tumour elimination [10, 11]. These studies demonstrated that distinct neutrophil phenotypes could influence the survival outcomes and response to immunotherapy in lung cancer. Although the NLR is an independent poor prognostic factor across multiple cancer types, the underlying mechanisms that regulate neutrophil infiltration and function within the TME is not well understood, especially in the early stages [12, 13]. It is plausible that different subpopulations of neutrophils with opposing functions dynamically expand and contract during tumour development. This raises the question of whether it is feasible to modulate the phenotype of neutrophils in early-stage lung cancer to improve immunotherapy efficacy and long-term patient outcomes.

**Fig. 1:**
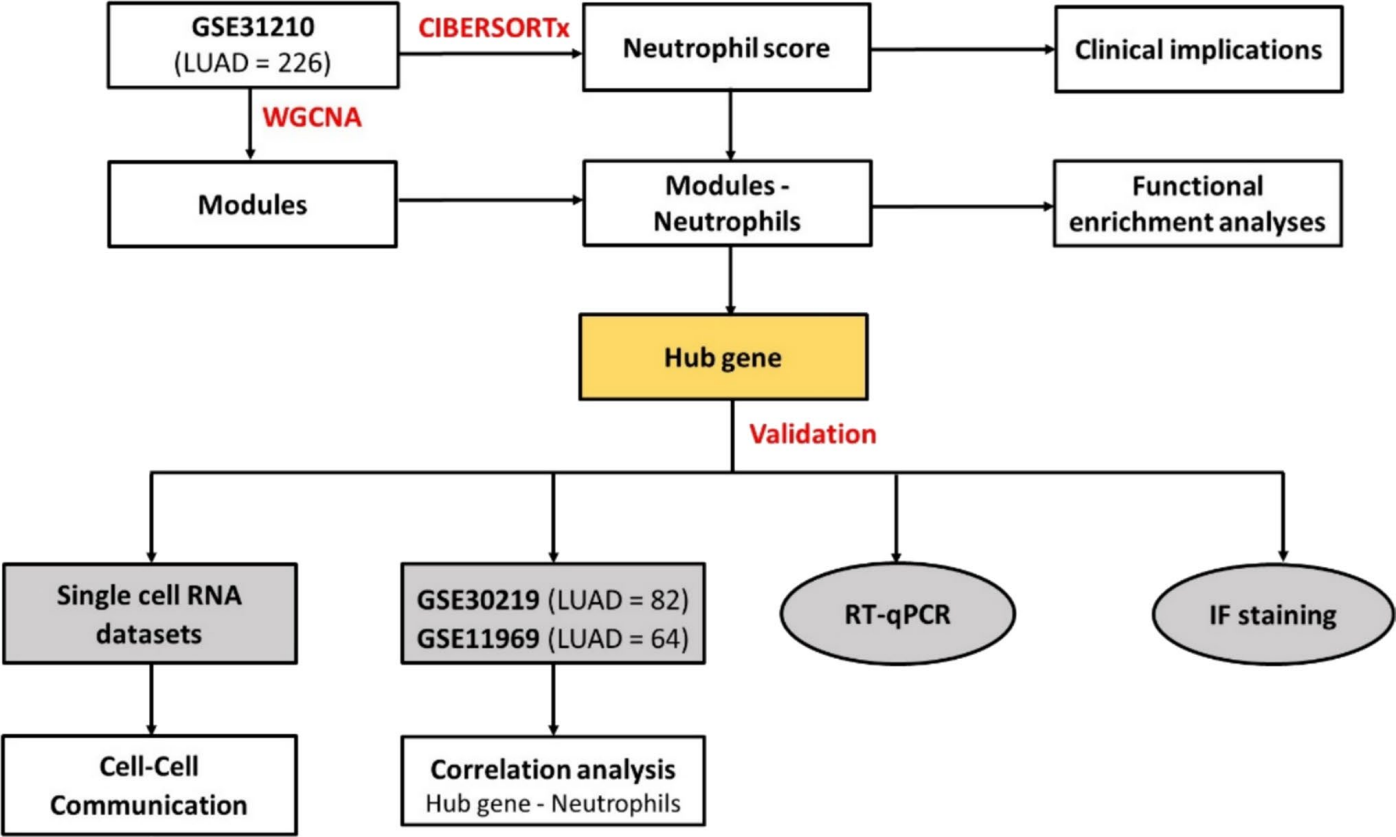
Flow diagram of the study design

In this study, we sought to interrogate publicly available transcriptomic datasets from early-stage LUAD tumour biopsy specimens. We used a combination of bioinformatic tools to unravel and validate key molecular targets that regulate neutrophils in early-stage LUAD (Fig. 1).

## Results

### Neutrophil infiltration in TME is associated with worse survival outcome, poorer response to immunotherapy, and higher risk of T-cell exclusion

**Fig. 2:**
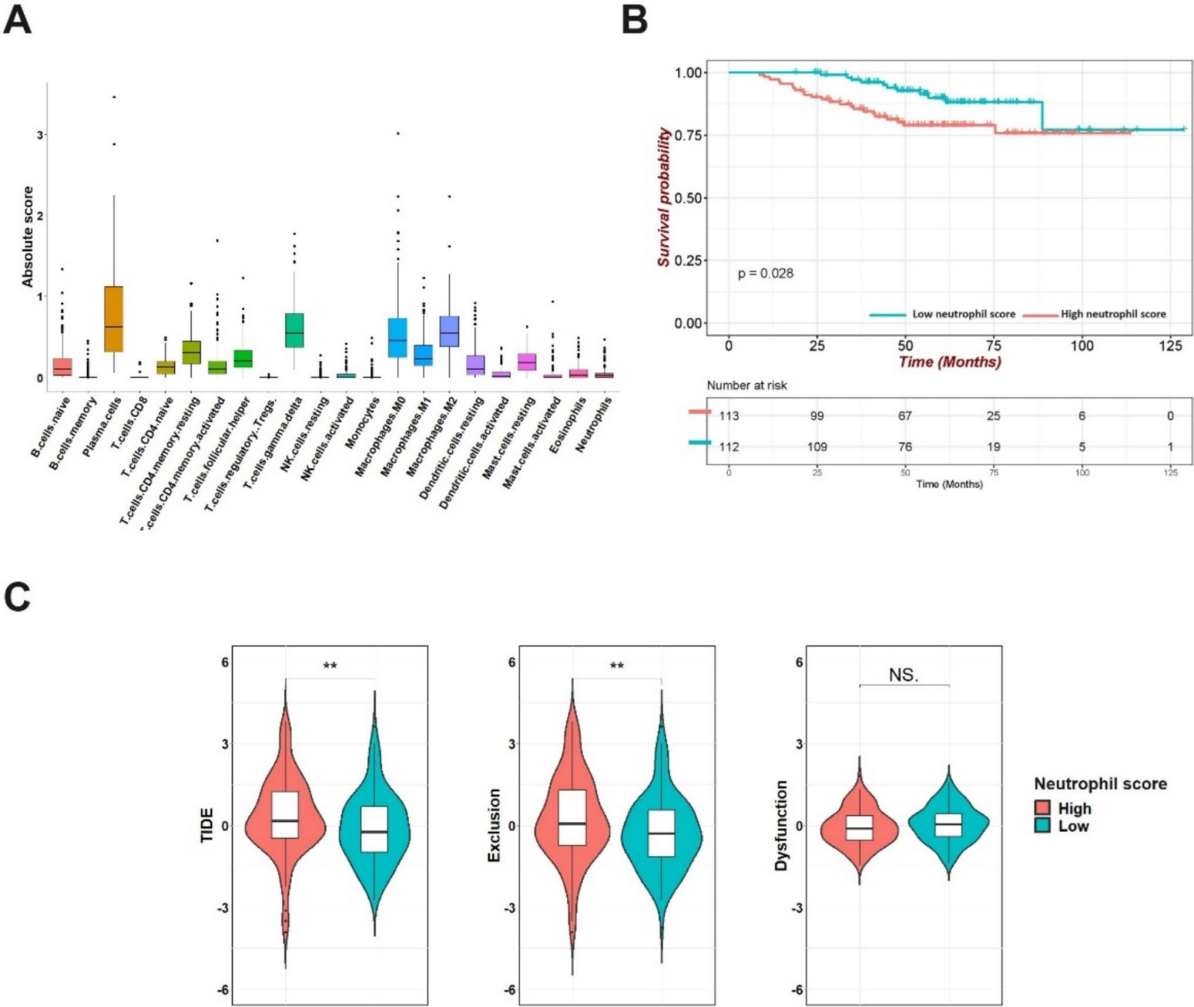
Neutrophils infiltration in early-stage LUAD tumour tissues (GSE31210) and the clinical implications. **A** Immune cell scores estimated by the CIBERSORT algorithm. Y-axis represents the absolute quantification **B** Kaplan–Meier survival analysis of patients stratified by neutrophil score **C** TIDE, T-cell exclusion, T-cell dysfunction scores were compared between the high and low neutrophil score groups (Kruskal–Wallis test. **p < 0.01, NS, no significance)

CIBERSORTx was used to assess immune cell abundance based on cell-type specific gene expression patterns from bulk transcriptomic profiling of early-stage LUAD samples. The absolute quantification (CIBERSORTx absolute score) of 22 innate and adaptive immune cells was calculated. The plasma cells and macrophages were relatively abundant **(**Fig. 2A**)**. Neutrophils were relatively low in abundance in early-stage LUAD. This is consistent with the literature using conventional (IHC staining) methods to detect TANs in tumour biopsies [9, 14]. Patients with high and low neutrophil score were then stratified by median value. In the KM survival analysis, a higher neutrophil score was associated with a significantly reduced overall survival (OS) rate in the GSE31210 dataset. This indicated that tumour-infiltrated neutrophils are skewed towards a pathogenic phenotype in this cohort **(**Fig. 2B**)**. We next evaluated whether the neutrophil score influenced cytotoxic T-cell functionality within the TME in this dataset, as immunosuppressive TANs are capable of blocking the function of cytotoxic T cells. We observed that the TIDE score was significantly higher in the high neutrophil group, suggesting that the TANs are phenotypically skewed towards an immunosuppressive state in this early-stage dataset **(**Fig. 2C**)**. Further analysis demonstrated that the neutrophil high group displayed a significant increase in the T-cell exclusion score but not T-cell dysfunction score. This suggested that TANs are likely to prevent the infiltration of cytotoxic T cells into the tumours **(**Fig. 2C**)**.

### Identification of hub gene correlated with neutrophil infiltration using WGCNA

**Fig. 3:**
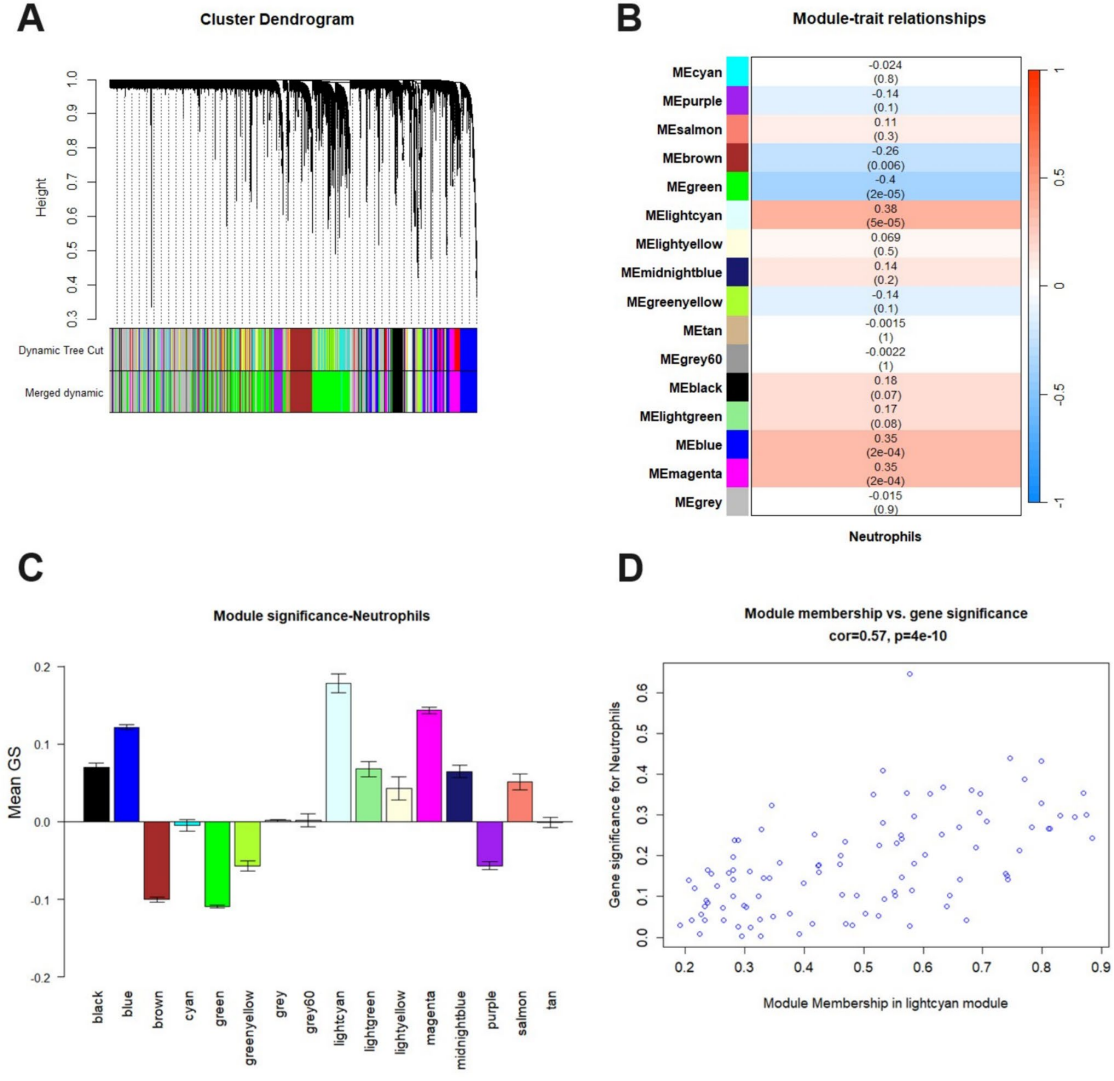
Weighted gene co-expression network analysis (WGCNA) results of GSE31210 dataset. **A** Dendrogram of input genes grouped into modules by hierarchical clustering. Modules of co-expressing genes are represented with different colours. **B** Module–trait relationship heatmap represents the correlation between gene modules and neutrophil score. The corresponding correlation coefficient and *p*-value are shown, with red indicates a positive correlation, blue indicates a negative correlation. **C** Distribution of mean gene significance (GS) among modules based on the correlation with neutrophil score. **D** Scatterplot shows the correlation between gene significance (GS) and module membership (MM) in the lightcyan module

To further explore the relationship between gene expression profile and neutrophil infiltration, gene co-expression networks (i.e. modules) were constructed by WGCNA. Using the hierarchical clustering method, highly interconnected genes were clustered into different modules coded with unique colours. A total of 16 modules containing 11,675 genes were identified after merging modules with highly correlated eigengenes as shown in the dendrogram **(**Fig. 3A**)**. Among them, the lightcyan module exhibited the highest positive correlation with neutrophil score (r = 0.38, *p* = 5e-05) **(**Fig. 3B**).** Moreover, the mean gene significance (GS) of the lightcyan module was the highest among all modules, which indicates most connections with neutrophil score were found in this module **(**Fig. 3C**)**. Finally, a scatterplot was generated for the lightcyan module, showing that Module Membership (MM) was highly correlated with Gene Significance (GS) (r = 0.57, *p* = 4e-10) **(**Fig. 3D**).** Therefore, the lightcyan module (N = 102 genes) was selected as the potential gene co-expression network correlated with neutrophil infiltration for downstream analysis.

**Fig. 4:**
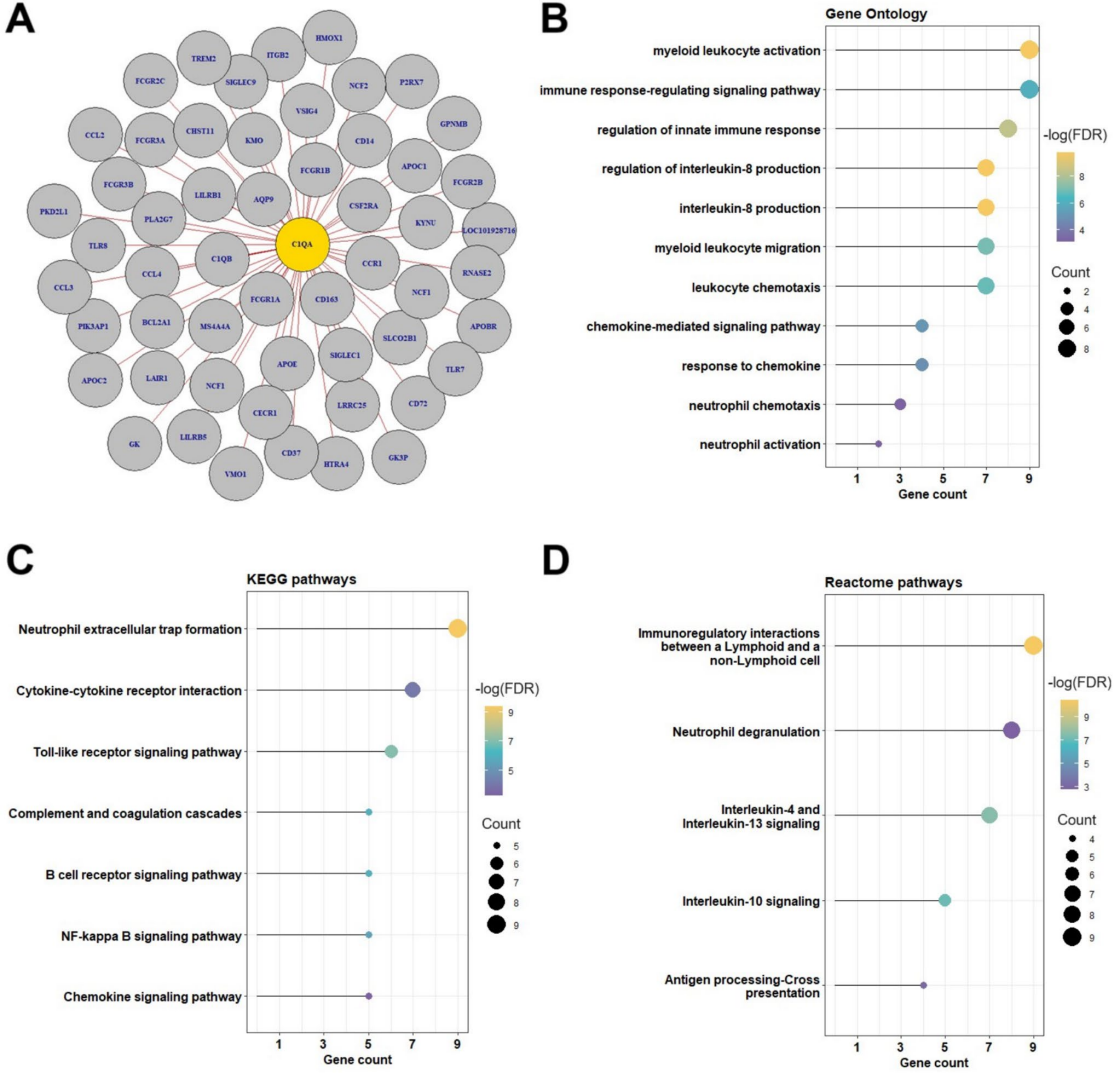
Hub gene identification and functional enrichment analyses of the lightcyan module. **A** Visualisation of gene network in lightcyan module (top 50 genes based on GS and MM). Genes in yellow bubbles are selected as hub genes. **B** Results of Gene Ontology (GO) analysis. **C** Kyoto Encyclopedia of Genes and Genomes (KEGG) pathway analysis. **D** Reactome pathway analysis

As shown in Fig. 4A, the lightcyan network is enriched with many neutrophil-related genes such as *FCGR3B (CD16), AQP9, CXCR2, and IL-8 (CXCL8),* which is a confirmation of WGCNA algorithm robustness. The functional enrichment analysis results suggested that the majority of intramodular genes are involved in neutrophil function, including neutrophil extracellular trap (NET) formation, neutrophil activation, neutrophil chemotaxis and neutrophil degranulation, immune response-regulating signalling, and chemokine-signalling pathways, interleukin-10 signalling **(**Fig. 4B, C and D**)**. Importantly, *C1QA* was identified as the hub gene with the highest connectivity within the lightcyan module, making it the primary molecular target for subsequent downstream analysis.

### External validation of WGCNA using publicly available datasets

**Fig. 5:**
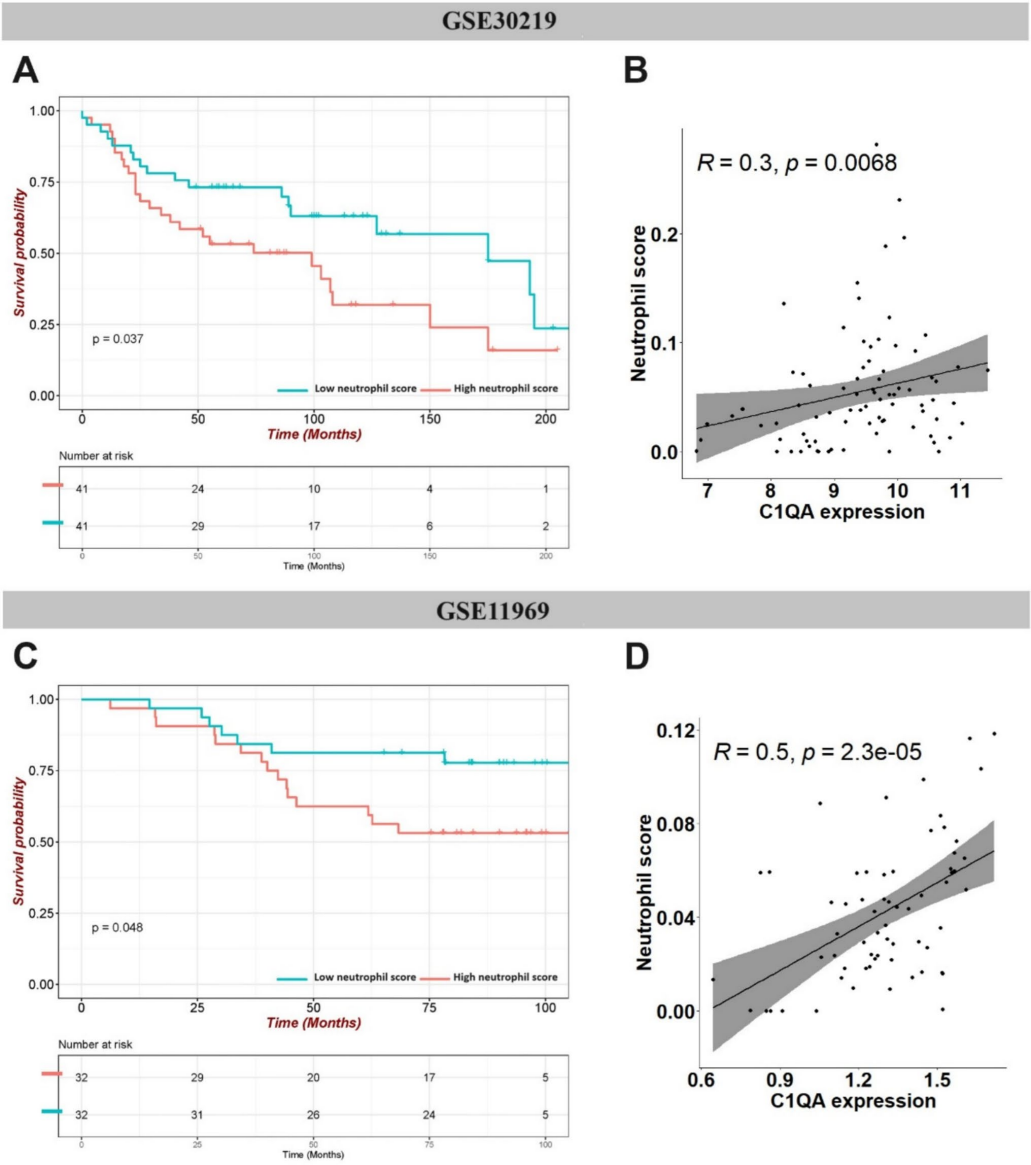
Expression levels of hub genes and neutrophil-associated verification by external databases. GSE30219 dataset: **A** Kaplan–Meier survival analysis of patients stratified by neutrophil score; **B** Scatterplots showing the Spearman’s correlation between the expression level of C1QA and neutrophil score; GSE11969 dataset: **C** Kaplan–Meier survival analysis of patients stratified by neutrophil score; **D** scatterplots showing the Spearman’s correlation between the expression level of C1QA and neutrophil score

To validate the WGCNA results, the correlation between *C1QA* expression and neutrophil abundance was examined using external datasets. Two independent GEO datasets of stages I–II LUAD samples were selected with higher neutrophil scores (calculated via CIBERSORTx) significantly associated with worse OS **(**Fig. 5A, C, Supplementary Table 1,2). Consistently, *C1QA* expression level showed positive correlation with neutrophil scores in these datasets (Fig. 5B, D). In addition, high C1QA expression was associated with poor prognosis in early-stage LUAD patients (Stages I–II) based on KM plotter analysis, further emphasising the critical role of this gene in tumour progression (Supplementary Fig. 1).

### C1QA potentially promotes immunosuppressive TAN accumulation in early-stage LUAD

**Fig. 6:**
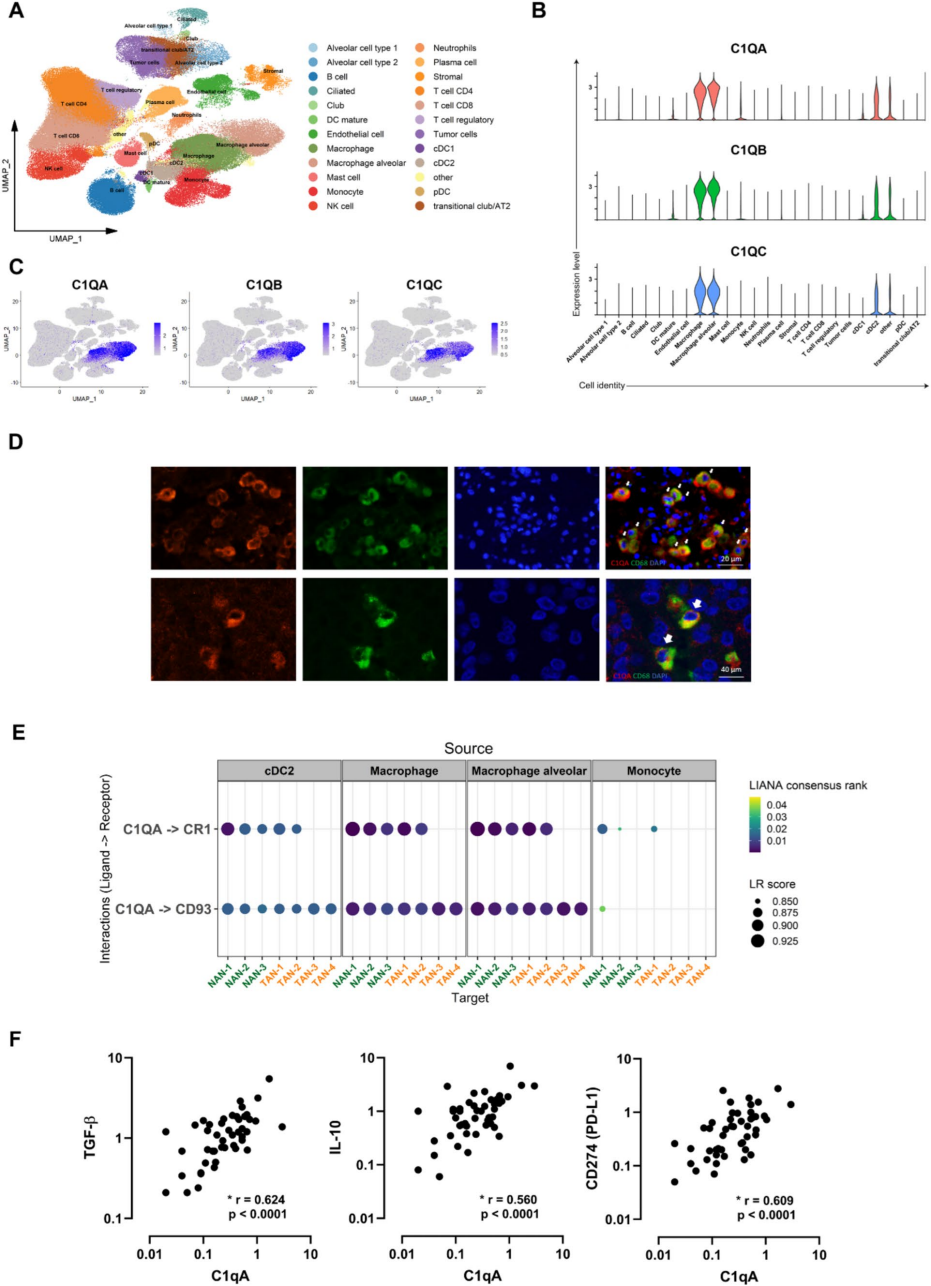
Macrophage-derived C1QA mediates efferocytosis that potentially promotes immunosuppressive neutrophils in TME **A** UMAP of early-stage LUAD tumour cells annotated by major cell types. **B** Violin plots showing the expression levels of *C1QA*, *C1QB*, and *C1QC* in different cell types. **C** Feature plots showing *C1QA*, *C1QB*, and *C1QC* distribution. The level of gene expression is indicated by colour intensity. **D** Representative IF staining images revealed co-localisation of C1QA and CD68 + macrophages in LUAD tissues. Red, C1QA; green, CD68; and blue, DAPI for nucleus. Scale bar 20 µm and 40 µm. **E** Cell–cell interaction dotplot showing L-R (Ligand–Receptor) interactions (LIANA consensus rank < 0.05) between ligand C1QA from different cells and its corresponding receptors in neutrophils. **F** Scatterplots representing the Spearman’s correlation between C1QA and IL-10, TGFB1, CD274 (PD-L1) relative gene expression (normalised to GAPDH) in the LUAD tumour samples

To further validate the bulk RNA-seq findings, we analysed scRNA-seq datasets of lung cancer from a previously published study [15]. The pre-processed Seurat objects (The single-cell lung cancer atlas (LuCA)—extended atlas) were downloaded and processed (https://cellxgene.cziscience.com/collections/edb893ee-4066-4128-9aec-5eb2b03f8287). Only stages I–II LUAD samples were included for cell–cell communication (CCC) analysis, consisting of 704,834 cells from 100 samples and 72 patients across 29 datasets. The cells were classified into 24 major cell types as described in the original study **(**Fig. 6A**)**. Neutrophils were further clustered into tumour-associated neutrophils (TANs) (TAN-1, TAN-2, TAN-3, and TAN-4) and normal adjacent tissue-associated neutrophils (NANs) (NAN-1, NAN-2, and NAN-3) [15].

C1QA, C1QB, and C1QC were most highly expressed in macrophages and alveolar macrophages, with moderate expression levels detected in Type-2 Conventional Dendritic Cells (cDC2) and other cells, as shown in Fig. 6B & C. The expression of C1QA in macrophages was confirmed with IF staining **(**Fig. 6D**)**. The LIANA pipeline was applied to explore the crosstalk patterns between neutrophils and different cells in the TME, potentially providing further insight into how our hub gene, C1QA, impacts tissue-resident neutrophils. Our CCC analysis revealed interactions between C1QA produced by different cells and its corresponding receptors (i.e. CR1 and CD93) on various neutrophil subsets **(**Fig. 6E**)**. Generally, the crosstalk of the ligand–receptor pair C1QA-CD93 was observed in all neutrophil subtypes, whereas communication from C1QA to CR1 appeared unique to NAN-1, NAN-2, NAN-3, TAN-1, and TAN-2. Given that our bulk RNA-seq findings indicated that C1QA modestly correlates with the abundance of neutrophils in the TME, these results indicate that C1QA does not regulate neutrophil recruitment but rather underscores its role in skewing neutrophils into an immunosuppressive state. On top of that, RT-qPCR results revealed the significantly positive correlations between C1QA and IL-10, TGF-β expression levels, highlighting the pro-tumour role of this hub gene in LUAD **(**Fig. 6F**)**. C1QA expression is also found to be positively associated with immune checkpoint molecule CD274 (PD-L1), a signature for T-cell exhaustion **(**Fig. 6F**).**

## Discussion

In this study, we first investigated the clinical implications of neutrophil infiltration in stages I–II LUAD tumour tissues. Our analysis on bulk RNA-seq and scRNA-seq datasets indicated that there is a relatively low abundance of neutrophils in early-stage LUAD biopsy specimens. Despite their relative low abundance, we found that neutrophil infiltration based on CIBERSORTx deconvolution analysis was associated with poorer survival outcomes across three separate GEO datasets. This indicates that in our identified early-stage LUAD cohorts, neutrophils were phenotypically skewed towards a tumour supportive phenotype, possibly by acting to suppress cytotoxic T-cell infiltration and functionality. Consistently, the previous studies have demonstrated that a higher proportion of TANs in the TME adversely impacted on tumour-infiltrating T lymphocyte (TIL) numbers through several mechanisms including (i) the formation of NETs that act as a physical tumour-surrounding barrier and (ii) the release of immunosuppressive cytokine secretion, which collectively desensitised tumour cells to immune checkpoint inhibitors (ICI) therapies (i.e. anti-CTLA-4, anti-PD-1, and anti-CD40 immunotherapy) [16, 17]. As a higher NLR has corresponded to poorer outcomes in both early and advanced solid tumours [18], our results further suggest that a high neutrophil infiltration rate is potentially a poor prognostic indicator in the early-stage LUAD. Complimentary with bulk RNA-seq findings, heterogeneous neutrophil subpopulations detected in the scRNA-seq datasets verified the co-existence of anti-tumour and pro-tumour neutrophil subsets in lung cancer [15]. While it is documented that neutrophils can have anti-tumour functions in early-stage NSCLC [11], the rate at which pro-tumorigenic neutrophils emerge with tumour progression will vary between individuals and consequently influence response to immunotherapies and the overall survival. Further work is needed to define distinct myeloid subsets throughout the development of solid tumours, as they may not only be prognostic, but may also reveal new therapeutic opportunities that work in a temporally sensitive manner.

WGCNA was then implemented to detect the key targets associated with neutrophil abundance in early-stage LUAD. The lightcyan module was identified as the gene co-expression network most closely associated with the neutrophil score, which consists of several molecules involved in neutrophil recruitment, polarisation, and function (e.g. *FCGR3B (CD16), AQP9, CXCR2, IL-8 (CXCL8), and IL-10)*. The functional enrichment analyses revealed that these co-expressing genes could orchestrate both innate and adaptive immunity through regulating the recruitment and function of neutrophils. Notably, many intramodular genes were enriched in the ‘NET formation’ pathway, which regulate the network of extracellular fibres that entrap pathogens for subsequent elimination. It is now recognised that NET pathways play a crucial role in immune suppression, cancer progression, metastasis, and anti-tumour therapy resistance [19, 20]. In addition, other enriched neutrophil-related pathways including ‘Regulation of interleukin-8 production’, ‘Interleukin-10 signaling’, and ‘Neutrophil activation’ highlighted the intricate interplay among intramodular genes to regulate neutrophil migration and function in the TME. Subsequently, *C1QA* (Complement Component 1 Q Subunit A) emerged as the central player in the lightcyan module. This suggests that this gene has an important influence on neutrophil populations in the TME. Therefore, we focused on elucidating the mechanisms by which this hub gene interacts with neutrophils.

C1q is a pivotal protein that belongs to the complement system. It primarily involves in the recognition and clearance of immune complexes, pathogens, and apoptotic cells [21]. C1q structurally is a tulip-like molecule, consisting of 18 polypeptide chains belonging to three subunits: A (28 kDa), B (25 kDa), and C (24 kDa) [22]. Emerging evidence from several studies suggests diverse functions for C1q in cancer context beyond the classical complement pathway activation [23, 24]. Notably, *Bulla *et al*. (2016)* reported retarded tumour growth and better survival in C1q-deficient (C1qa − / −) mice from an in vivo syngeneic B16 melanoma model. This study highlighted the pro-tumour role of C1q in cancer [23].

In this study, we identify C1QA as a macrophage-derived mediator modestly associated with neutrophil abundance but were prognostic of poor survival outcomes in early-stage LUAD. Our cell–cell communication analysis confirms that C1QA interacts with neutrophils via its well-characterised complement receptors [21, 26, 27] including CR1 (CD35) and CD93, and this interaction may influence the neutrophil phenotype. Although C1q is known to enhance efferocytosis by acting as a pattern recognition receptor and engaging phagocyte receptors [25], its specific role in neutrophil function within the TME remains underexplored. Notably, our in silico scRNA-seq analysis indicated that these C1QA–receptor interactions were more prominent in neutrophil subsets not previously associated with tumour promotion [15], suggesting a possible context-specific role. While efferocytosis has been linked to immunosuppressive reprogramming and anti-inflammatory cytokine production in other myeloid cells [28–30], direct evidence for this mechanism in neutrophils within LUAD is limited [26]. Nonetheless, our observed correlation between C1QA, IL-10, and TGFB1 supports a broader role for C1QA in shaping an immunosuppressive TME. Further studies are required to determine whether neutrophils contribute to immunosuppressive processes through efferocytosis, which is supported in the previous studies [31] [32], or alternative pathways. Finally, we acknowledge that our study is limited to early-stage LUAD, and future investigations involving advanced disease and functional validation will be essential to fully elucidate C1QA’s role in neutrophil regulation.

## Conclusions

Our study identifies C1QA as a poor prognostic marker for early-stage LUAD. As neutrophils are increasingly recognised as important regulators in lung cancer, we suggest that C1QA–neutrophil interactions may promote the emergence of immunosuppressive TANs, supporting a pro-tumour microenvironment in early-stage LUAD. Targeting C1QA and its regulatory pathway may, therefore, represent a promising therapeutic strategy in cancer.

## Materials and methods

### Data acquisition and processing

Gene expression data and clinical information were obtained from GSE31210 dataset consisting of 226 patients with stages I–II LUAD (GEO database: https://www.ncbi.nlm.nih.gov/geo/). This dataset served as a discovery cohort in this study. Patient information is detailed in Table 1**,** and the study design is summarised in Fig. 1.

**Table 1:** Patient characteristics from GSE31210 dataset

Characteristics	Variables	Number of samples (*N* = 226)
Gender	Female	121 (53.5%)
	Male	105 (46.5%)
Age at inclusion (year)	< 60 years	96 (42.5%)
	≥ 60 years	130 (57.5%)
Stage	I	168 (74.3%)
	II	58 (25.7%)
Smoking status	Yes	111 (49.1%)
	No	115 (50.9%)

In addition, two independent datasets of stages I–II LUAD, namely GSE30219 (n = 82) and GSE11969 (n = 64), were downloaded and analysed as independent validation cohorts. Patient details are provided in Supplementary tables 1 and 2, respectively.

### Evaluation of immune cell landscape

The CIBERSORTx algorithm was applied to determine the absolute quantification of 22 types of immune cells in LUAD samples from the GSE31210 dataset. CIBERSORTx is an in silico analytical tool that is widely used to deconvolute immune cell populations from gene expression data [33]. Absolute score indicates the absolute proportion of immune cells in a mixture, where higher scores indicate a higher proportion of distinct immune cells. CIBERSORTx results with a *p*-value < 0.05 were considered reliable to be used for further analysis. Kaplan–Meier (KM) survival analysis was performed to explore the prognostic value of the neutrophil score.

### Tumour immune dysfunction and exclusion (TIDE) score

TIDE score (http://tide.dfci.harvard.edu/), a combination of the T-cell dysfunction estimated from hot tumours and the T-cell exclusion estimated from cold tumours, was used to evaluate the impairment of the immune system and to predict the response to immunotherapy between high and low neutrophil score groups [34]. Higher TIDE score indicates greater risk of immune evasion and worse response to immunotherapy. In addition, T-cell Dysfunction score and T-cell Exclusion score were also assessed separately.

### Weighted gene co-expression network analysis (WGCNA)

Top 50% most variable genes (11,675 genes) from GSE31210 dataset were used as input for WGCNA algorithms [35] to explore the key genes associated with neutrophil infiltration (i.e. neutrophil score) in the early-stage LUAD. Briefly, a similarity matrix was constructed and then converted into an adjacency matrix, followed by the transformation into the topological overlap matrix using a suitable soft threshold power. The minimum module size cut-off was set at 30 genes, and gene modules with ≤ 40% dissimilarity were merged. Correlation analysis was conducted between the gene modules and neutrophil score. Module with the strongest positive correlation and *p*-value < 0.05 was selected; and hub gene is identified as the gene with the highest connectivity using ‘chooseTopHubInEachModule’ function of WGCNA. Visualisation of gene module network was conducted with ‘igraph’ R package (https://cran.r-project.org/web/packages/igraph/index.html).

### Functional enrichment analysis

Functional enrichment analysis is important to understand the biological characteristics of transcriptome data. Gene Ontology (GO), Kyoto Encyclopedia of Genes and Genomes (KEGG), and Reactome pathway databases from the ‘clusterProfiler’ (version 4.8.0) and ‘ReactomePA’ (version 1.44.0) R packages [36, 37] were used to confirm the association of the selected module with neutrophil-related functions. Enriched terms with *p*-value < 0.05 and false discovery rate (FDR) < 0.05 were considered significant.

### External validation of the hub genes

Two external datasets, GSE30219 (n = 82) and GSE11969 (n = 64), were used to validate the correlation of the hub genes with neutrophil scores (calculated with CIBERSORTx). KM survival analysis was also performed to explore the prognostic value of neutrophil score in these datasets.

The association of *C1QA* expression level with the overall survival in a merged dataset of early stage LUAD cases was evaluated using ‘KM plotter' database (https://kmplot.com/analysis/) [38]. The KM Plotter integrates gene expression and clinical data from multiple sources, including the Gene Expression Omnibus (GEO), European Genome-phenome Archive (EGA), and The Cancer Genome Atlas (TCGA), making it a comprehensive platform for biomarker discovery and prognostic evaluation.

### Cell–cell communication (CCC) analysis

LIANA (version 0.1.13) [39], a tool integrating multiple methods for Cell–cell communication (CCC) inference in single-cell data, was used to explore cell–cell ligand–receptor (L-R) interactions of different cells with neutrophil subtypes from early-stage LUAD datasets (https://cellxgene.cziscience.com/collections/edb893ee-4066-4128-9aec-5eb2b03f8287) [15]. LIANA provides a consensus-based rank aggregate for L-R pairs from the results of multiple CCC pipelines through ‘robust rank aggregation’ (RRA). In this study, L-R pairs were inferred on the normalised gene expression data using five different CCC methods (SCA, NATMI, Connectome, CellPhoneDB, and CytoTalk). L-R interactions were further ordered and filtered based on RRA < 0.05, and the expression magnitude for each L-R pair based on SingleCellExperiment’s LR Score was also reported.

### Human ethics approvals for resection biopsy cohort

LUAD tumour tissues excess to diagnostic requirements were obtained from the Victorian Cancer Biobank from NSCLC patients undergoing resection surgery at the Royal Melbourne Hospital (https://viccancerbiobank.org.au/). Ethics approval was also obtained from RMIT University, Australia (Ethics ID: SEHAPP 09–17), in accordance with the Declaration of Helsinki. Informed consent was obtained from all subjects and/or their legal guardian(s).

### Real-time quantitative PCR (RT-qRCR)

Total RNA was isolated from snap frozen resection specimens using the AllPrep DNA/RNA/miRNA Universal kit (Qiagen) as previously described [40, 41]. Total RNA was converted to cDNA by using the Superscript IV VILO kit (Thermo Fisher Scientific). Quantitative PCR was performed on the QuantStudio 7 (Applied Biosystems, Foster City, CA) using validated TaqMan primers formatted in a TaqMan Low-Density Array. The threshold cycle values were normalised to the geometric mean of the reference housekeeping gene GAPDH. The relative mRNA expression was calculated in triplicate using the 2-ΔΔCt method.

#### Immunofluorescent (IF) staining

Immunofluorescent (IF) staining was performed as previously described [40]. Primary antibodies against CD68 (1:100, DAKO, #M0814) and C1QA (1:100; Thermo Fisher Scientific, #PA5-29,586) were used. The slides were subsequently incubated with secondary antibodies, namely donkey anti-mouse IgG/FITC (1:100; Thermo Fisher Scientific, #A32766) and goat anti-rabbit IgG/TRITC (1:100; Thermo Fisher Scientific, #A21428). Images of C1QA and CD68 positive cells were captured at low- (× 20) and high-power (× 40) magnification on the microscope (Olympus, Tokyo, Japan).

#### Statistical analysis

All data analysis and visualisation were conducted using R version 4.3.0 and GraphPad Prism (version 10.1.2). *P*-value < 0.05 was considered statistically significant.

## Supplementary Information

Below is the link to the electronic supplementary material.

Supplementary file1 (DOCX 261 KB)

## Data Availability

No datasets were generated or analysed during the current study.
