# Supplementary material for: Identification of C1QA as a prognostic marker and regulator of immunosuppressive neutrophils in early-stage lung adenocarcinoma through integrated bioinformatics analyses
Source: Clin Exp Med. 2025 Oct 31;25(1):348. doi: 10.1007/s10238-025-01881-y (PMC12578707; doi:10.1007/s10238-025-01881-y)
Supplement: Supplementary file 1 — Supplementary file1 (DOCX 261 KB) [file 10238_2025_1881_MOESM1_ESM.docx]

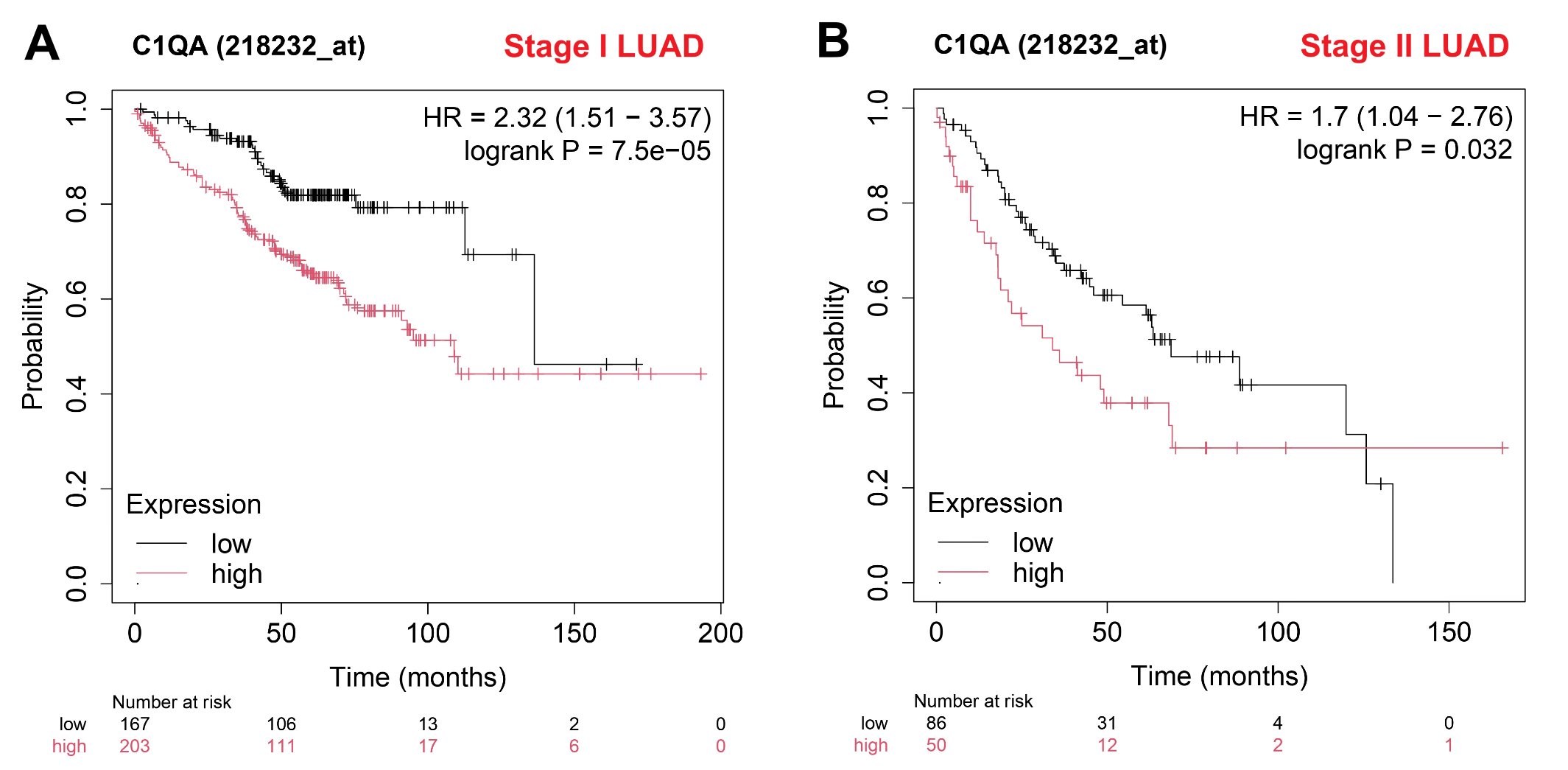


**Supplementary figure 1. The prognostic value of C1QA transcriptional level in early-stage LUAD cohort**. C1QA expression level and overall survival rate in (A) Stage I LUAD patients (N = 370) (B) Stage II LUAD patients (N = 136)

**Table S1. Patient characteristics from GSE30219 dataset**

| Characteristics | Variables | Number of samples (N=82) |
| --- | --- | --- |
| Gender | Female | 18 (22%) |
|  | Male | 64 (78%) |
| Age at inclusion (year) | <60 years | 36 (43.9%) |
|  | ≥60 years | 46 (56.1%) |
| Stage | I | 70 (85.4%) |
|  | II | 12 (14.6%) |

**Table S2. Patient characteristics from GSE11969 dataset**

| Characteristics | Variables | Number of samples (N=64) |
| --- | --- | --- |
| Gender | Female | 27 (42.2%) |
|  | Male | 37 (57.8%) |
| Age at inclusion (year) | <60 years | 20 (31.3%) |
|  | ≥60 years | 44 (68.7%) |
| Stage | I | 51 (79.7%) |
|  | II | 13 (20.3%) |
| Smoking status | Yes | 33 (51.6%) |
|  | No | 31 (48.4%) |
